# Massive blood loss protocol ‘Code Red’ at Papworth Hospital: A closed loop audit

**DOI:** 10.1177/1750458920943361

**Published:** 2020-09-08

**Authors:** M Tennyson, J Redlaff, G Biosse-Duplan, M Lewin, N Jones, H Layard Horsfall

**Affiliations:** 1 Addenbrooke’s Hospital, Cambridge, UK; 2 School of Clinical Medicine, University of Cambridge, Cambridge, UK; 3 Department of Transfusion, Addenbrooke’s Hospital, Cambridge, UK; 4 Department of Cardiothoracic Anaesthesia and Critical Care Medicine, Papworth Hospital NHS Foundation Trust, Cambridge, UK; 5 Division of Neurosurgery, Department of Clinical Neurosciences, Addenbrooke’s Hospital and University of Cambridge, Cambridge, UK

**Keywords:** Massive blood loss, Intensive care, Transfusion, Cardiothoracic, Haemorrhage protocol transfusion, Massive haemorrhage, Audit, Quality improvement, Cardiothoracics

## Abstract

**Aim:**

To investigate if the massive blood loss protocol ‘Code Red’ at a specialist cardiothoracic hospital was activated according to local and national guidelines by a closed loop audit.

**Methods:**

Electronic and paper patient care systems were searched in 2015 and 2018 to access records for the ‘Code Red’ activations. Activation of the massive blood loss protocol was compared against the national standards set by The British Committee for Standards in Haematology. The percentage of cases meeting each of the ten standards in the specialist cardiac unit’s Protocol for the Management of Massive Blood Loss in Adults (adapted from the national standards) were evaluated.

**Results:**

‘Code Red’ protocol was activated on 18 occasions in 2015 and nine occasions in 2018, representing just 0.83 and 0.26% of emergency surgeries, respectively. Between 2015 and 2018, there was a 6% increase of ‘Code Red’ cases being appropriately activated, a 26% increase in the prompt notification of the haematology department upon activation, alongside a 30% increase in the timely delivery of blood products, and a 25% decrease in the average amount of blood transferred prior to ‘Code Red’ activation.

**Conclusion:**

There has been an improvement in the standards of care and management of massive blood loss this specialist cardiac centre despite the target timeframe being reduced from 30 to 15min between 2015 and 2018. Preparation for and anticipation of massive blood loss has likely decreased the number of incidences requiring ‘Code Red’ activation, permitting delivery of safe patient care.

**Provenance and Peer review:** Unsolicited contribution; Peer reviewed; Accepted for publication 29 June 2020.

## Introduction

*Our institution* is an international centre of excellence in cardiothoracic surgery, specifically reparative and transplant, and intensive care. Blood loss and transfusion are often associated with cardiac surgery due to the invasiveness into the circulatory system, in addition to the effect of cardiopulmonary bypass on platelet function and associated coagulopathy (Kehara et al 2014, Paparella et al 2004). Further, the need for prophylactic high-dose anticoagulation increases the risk of massive blood loss. Massive blood loss is a serious clinical situation associated with worse patient outcomes, including a higher mortality (Stainsby et al 2006b).

Guidelines from the British Committee for Standards in Haematology (BCSH) (Hunt et al 2015, Stainsby et al 2006b) and the Association of Anaesthetists of Great Britain and Ireland (Thomas et al 2010) stress the need for effective recognition of haemorrhage and an effective protocol. Designing a protocol requires a definition of severe haemorrhage based on parameters monitored in surgery as opposed to using retrospective measures. The BCSH guidelines define massive blood loss as a bleed that leads to a heart rate of more than 110beats/min and/or systolic blood pressure less than 90mmHg (Hunt et al 2015), though hospitals may have individually mandated criteria based on local expertise and experience. Haemorrhage is a critical situation and blood transfusion is a necessary part of treatment; however, it is not without risks (Koch et al 2006). Adverse outcomes stem from the known risks of transfusion such as transfusion-related acute lung injury, transfusion-related immunomodulation, infection, and dilution coagulopathy. It was reported that the most common adverse event following transfusion is a haemolytic reaction caused by the giving of the wrong blood to the patient (Stainsby et al 2006a). Moreover, evidence shows that there is no difference in composite end point (death from any cardiovascular cause) between a restrictive and a liberal transfusion strategy (Hajjar et al 2010, McIntyre et al 2004, Mazer et al 2017). This finding highlights the need for efficient protocols that must be followed, particularly in acute and emergency medicine. While massive haemorrhage is in itself a risk factor for serious postoperative complications and death, transfusions carry their own independent risks and thus protocols must be installed, followed, and activated only at the appropriate times. Protocolisation in addition permits evaluation of the treatment strategy.

The BCSH 2006 and 2015 (Hunt et al 2015, Stainsby et al 2006b) guidelines on massive blood loss management outline parameters that must be kept within appropriate ranges and indicate which blood cell components must be used to do so. The guidelines stress the need for efficient communication between the clinical teams and blood transfusion laboratories, as well as the need for anticipation of scenarios that may require thawing or delivery of blood products. This inevitably requires thorough protocols.

The specialist cardiac unit has its own protocol on massive blood loss in adult cardiothoracic surgery, the unit's Protocol for the Management of Massive Blood Loss in Adults (‘Code Red’) DN526, based on the BCSH 2006 and 2015 guidelines. The protocol aims to:

1. Maintain blood volume ensuring tissue oxygenation
2. Control bleeding by surgical intervention and use of blood component therapy to stem the bleeding
3. Prevent secondary haemodilution

Activation of the ‘Code Red’ protocol leads to distribution of ‘Pack A’, containing 4 units of red blood cells (RBCs) and fresh frozen plasma (FFP) and one unit of platelets. This mobilisation of blood products is done rapidly and does not require blood test results or the haematologists’ consent.

We aimed to audit when ‘Code Red’ is activated appropriately, according to the hospital-outlined criteria defining massive blood loss. This was achieved via a full-cycle, closed loop audit in 2015 and 2018. The implications of this are two-fold: reducing morbidity and reducing cost. Perioperative transfusion (regardless of number of units or type of blood product) increases postoperative mortality and risk of infection (Horvath et al 2013, Koch et al 2006); thus, it follows that limiting transfusion frequency to only when necessary not only improves patient outcomes but also reduces treatment costs.

## Methods

A closed loop audit cycle was performed on the appropriateness of ‘Code Red’ activation according to hospital guidelines at the specialist cardiac unit, to assess changes made since the initial audit in 2015.

The Laboratory Information System (electronic patient records) was searched for ‘Code Red’ to establish the number of times that ‘Code Red’ protocol was activated in the 12 months of 2015. The results were then cross-checked against paper request forms at the haematology laboratory. To assess whether the protocol was activated appropriately, we established the amount of blood loss prior to activation of ‘Code Red’ by searching the relevant patient care systems: SaferSleep and Metavision (anaesthetic in-theatre records), and CIS (Intensive Care Unit records). For each standard set by the BCSH guidelines, the percentage of cases in which that standard was met was calculated. One unit of RBCs was assumed to be equivalent to 250–350ml.

The initial audit data collected in 2015 was analysed and clinical stakeholders at the specialist cardiac unit were informed of the results with the intention of emphasising both awareness of ‘Code Red’ protocol and importance of accurate and correct documentation of steps within the protocol. Data collection (as above) was repeated for the 12 months of 2018 and activation was compared against the standards (The cardiac unit's Protocol for the Management of Massive Blood Loss in Adults (‘Code Red’) DN526), with four standards added to reflect the update of the 2015 BCSH guidelines for the Haematological Management of Major Haemorrhage (Hunt et al 2015).

## Data collection

During the 2015 audit cycle, there was absence of documentation on the digital systems for six activations and we were unable to locate the paper request forms. These six activations were excluded from the data analysis for 2015. There were no data missing from the 2018 cycle.

## Results

### Patient demographics

There were 18 and nine patients in the 2015 and 2018 cohorts, respectively (Table 1). The mean age in 2015 was 57.9 years, a male:female ratio of 14:4, and BMI of 29.0. The 2018 cohort had a mean age of 57.3 years, a male:female of 5:4, and a BMI of 22.8.

**Table 1:** Summary of ‘Code Red’ activation patient cohort demographics for 2015 and 2018

Patient demographics	2015	2018
Mean age (years)	57.9	57.3
Male:female	14:4	5:4
Mean weight (kg)	88	70.1
Mean BMI	29.0	22.8
Total patients (n)	18	9

BMI: body mass index (kg/m^2^).

In 2015, 22% of ‘Code Red’ patients were undergoing an elective operation, whereas this increased to 56% of the cohort in 2018 (Table 2). Conversely, ‘Code Red’ patients undergoing emergency surgery made up 67% of the cohort in 2015; however, only 33% of the 2018 cohort. The proportion of ‘Code Red’-activated patients undergoing each type of surgical procedure was different between the cohorts. In the 2015 cohort, 67% of patients were undergoing cardiac surgery, 17% thoracic, and 17% transplant, whereas in 2018, the surgeries consisted of 45% cardiac, 22% thoracic, 22% transplant, and for one patient (11%) the type of surgery is unknown.

**Table 2:** Context of ‘Code Red’ activations in 2015 and 2018

‘Code Red’ activation	2015	2018
Total patients (n)	18	9
Admission		
Immediately postoperative	13	8
Transplant	2	0
Cardiac (non-transplant)	1	0
ECMO (no surgery)	1	1
Priority of surgery		
Emergency	12	3
Elective	4	5
Unknown	2	1
Type of surgery		
Cardiac	12	4
Thoracic	3	2
Transplant	3	2
Unknown	0	1

ECMO: extracorporeal membrane oxygenation.

### Patient outcomes 24h after ‘Code Red’ activation

Of the 18 patients in our 2015 cohort, 13 (72%) recovered in the intensive care unit (ICU), one patient (6%) died within 24h of ‘Code Red’ activation. A further three patients (18%) died in the subsequent postoperative days (Table 3). In 2018, seven patients (78%) recovered in ICU, whereas two (22%) died during the 24h post-protocol. One patient who died had been admitted to the cardiac unit for extra-corporal membrane oxygenation following multiorgan failure of an unknown aetiology and died in theatre. The second patient was admitted for pneumonectomy but returned to theatre within 12h of the first operation for re-exploration given suspected bleeding. ‘Code Red’ was activated postoperatively on return to the ward; however, the patient died before blood could be transfused.

**Table 3:** Patient outcomes after ‘Code Red’ activation in 2015 and 2018

Patient outcomes	2015	2018
Recovered in ICU	13	7
Deaths within 24h	1	2
Deaths within one week	3	0
Unknown	1	0
Total patients (n)	18	9

ICU: intensive care unit.

### Criteria for massive blood loss

Comparison of the percentage of cases in which each standard was achieved is shown in Table 4. The average amount of blood transfused prior to activation reduced from 10.1 units in 2015 to 7.6 units in 2018, and our results show that on re-audit ‘Code Red’ was activated in line with protocol 89% of the time, an improvement of 6%. We also noticed an increase of 26% in the proportion of cases in which haematology consultants were informed of the ‘Code Red’ activation (and thus their ability to prepare Pack B for request in the event of continued bleeding), and a 30% increase in the timely receipt of blood products after ‘Code Red’ activation. We note that in 56% of cases in 2018, blood products were received in under 10min (Table 5). The use of the minimum required number of blood products decreased by 66.6%, however.

**Table 4:** The percentage of ‘Code Red’ activations in 2015 and 2018 that met each of the ten standards in the unit's Protocol for the Management of Massive Blood Loss in Adults

Standard	Description	Cases meeting standard (n, %)	
		2015	2018
I	Major blood loss condition fulfilled before activation of ‘Code Red’	83.3	88.9
II	Haematology informed in under 10min	54.5	80
III	Products received within 30min of ‘Code Red’ activation	45.5	75
IV	Transport and transfusion within 15min once products are ready^a^	40	50
V	Minimum number of products issued	100	33.3
VI	If patient is on bypass, platelets should not be issued in Pack A	62.5	100
VII	Hb maintained above 80 after transfusion	^b^	57.1
VIII	Acidosis actively managed to keep base excess above −5	^b^	71.4
IX	Further dose of TXA after ‘Code Red’ activation	^b^	14.3
X	Samples sent for FBC and coagulation after every 3–5 units of blood transfused	^b^	85.7

FBC: full blood count; Hb: haemoglobin; TXA: tranexamic acid.

^a^Standard IV – time was reduced from 30 to 15min due to labs carrying two defrosted units of FFP.

^b^Standards VII–X were not part of the 2015 audit and added to the unit's Protocol in response to the 2015 update of the BCSH guidelines.

**Table 5:** Number of units of red blood cells transfused prior to ‘Code Red’ activation and the period of time, in minutes, from activation to products being received, in the 2018 cohort. A unit of red blood cells was defined as 250–350ml

Patient	RBC excluding cell save (units)	‘Code Red’ activation to products received (min)
1	4	39
2	7	40
3	10	4
4	8	5
5	10	15
6	0	11
7	5	4
8	12	7
9	12	4
Mean	7.56	14.33

RBC: red blood cell.

## Discussion

The aim of the audit was to evaluate whether criteria for ‘Code Red’ activation were met, and whether our 2015 audit affected appropriateness of protocol activation. We found that the specialist cardiac unit performed well, with the protocol appropriately activated 89% of the time in 2018. There was also an improvement in timely product preparation: products distributed within 30min from ‘Code Red’ activation improved dramatically from 46 to 75%, despite the change in audit standards in 2018 to a new goal of having products ready within 15min (Table 4). This result might reflect the systematic improvements which were introduced after first audit cycle, such as a new process ensuring two bags of pre-thawed plasma had to be kept in the lab. This has increased the speed at which Pack A is made available by the transfusion practitioners at the specialist cardiac unit.

**Figure 1:**
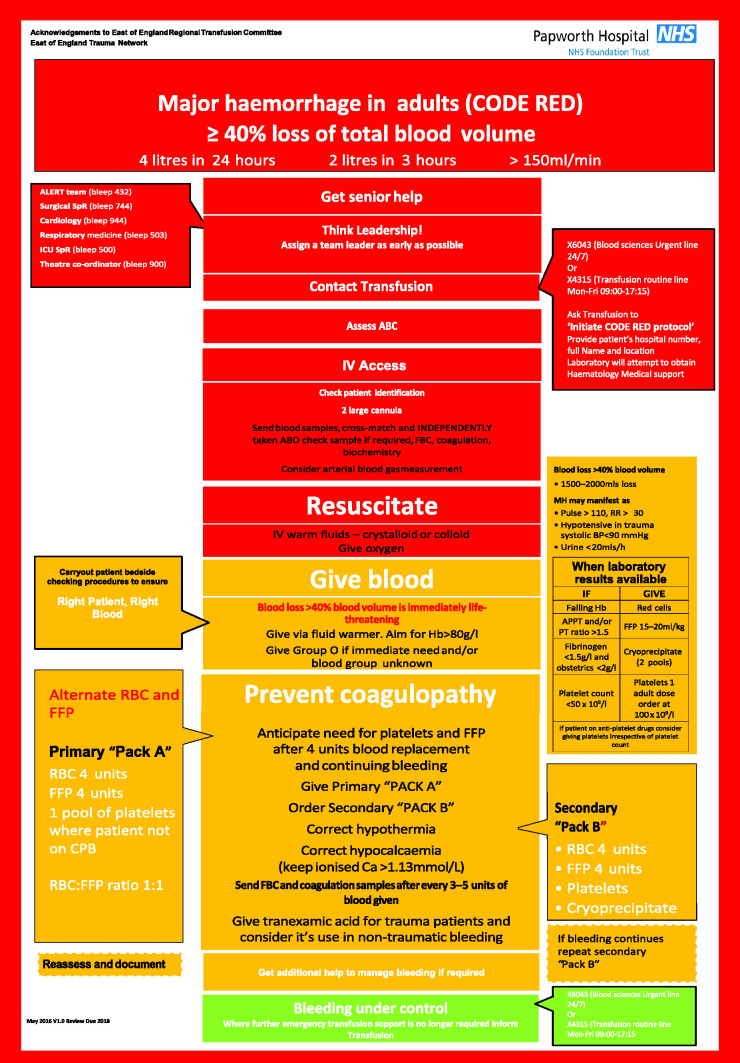
‘Code Red’ Protocol APPT: ; BP: ; FBC: full blood count; FFP: fresh frozen plasma; ICU: intensive care unit; IV: ; MH: ; PT: ; RBC: red blood cell; RR: .

As expected due to appropriate planning prior to surgery, there are lower rates of ‘Code Red’ activation in elective operations than emergency operations for both audited years (2015 and 2018). In 2015, at the specialist cardiac unit there were a total of 1442 emergency surgeries and 2878 elective surgeries performed (Table 6). ‘Code Red’ protocol was activated in 0.83% of emergency surgeries but only in 0.14% of elective surgeries. The total number of surgeries remained similar in 2018, with 1144 emergency and 2897 elective surgeries performed; however, the proportion of ‘Code Red’ activation changed to 0.26 and 0.17%, respectively. Preparation for surgery involves ordering appropriate blood products as well as patient optimisation in the case of any known coagulopathies (such as haemophilia). The high standards of care at the cardiac unit involving meticulous pre-planning in anticipation of massive blood loss also potentially explains the low number of ‘Code Red’ activations, which could possibly be a gross underestimate of massive blood loss occurrence. Indeed, after presentation of our 2015 audit cycle to the department, it was decided that the specialist cardiac unit would move towards individual clinicians’ discretion to activate the protocol. This flexibility and preparation should be seen as an encouraging finding, indicating that forward planning and excellent haematologist support limits the need for the activation of such a protocol as timely supply of blood products and expert advice is still readily available. In 2018, only three of the nine patients (standard V, Table 4) had a ‘classic’ Pack A (RBCs:FFP:platelets in the ratio 4:4:1) issued (Figure 1). The remaining patients all had customised packs, with one patient having only two cryoprecipitate and one pack of platelet issued in Pack A. This patient-specific response accounts for why the standard was only met in 33% of activations. The reduction in RBC pack transfusion and increased ‘personalised transfusion’ is supported by high-quality evidence: an observational cohort study of 11,963 patients undergoing cardiac surgery found that RBC transfusion increased the risk of all postoperative adverse events, and that morbidity increased incrementally with each additional RBC unit transfused (Koch et al 2006). Two randomised controlled trials in which RBC transfusion protocol was purposefully triggered at more restrictive thresholds found that increasingly strict thresholds did not negatively impact patient outcomes when compared to cases where a liberal approach to transfusion was used (Hajjar et al 2010, Mazer et al 2017). Furthermore, the ratio itself of blood products transfused has been seen to affect outcomes: a higher plasma:RBC ratio was shown to lower mortality and organ dysfunction (Delaney et al 2017), and transfusion of a 1:1:1 ratio of plasma:platelets:RBCs led to faster haemostasis than a 1:1:2 ratio (Holcomb et al 2015). As well as adverse cardiovascular effects, another study demonstrated that each RBC unit also increases crude risk of infection by 29% (Horvath et al 2013).

**Table 6:** Total number of surgeries at the specialist cardiac unit in 2015 and 2018

	Number of emergency surgeries	Number of elective surgeries
2015	1442	2878
2018	1144	2897

Our audit of the ‘Code Red’ guidelines is most limited by the small sample size and incomplete data which, in addition to the complexity of these patients, prevents definitive conclusions on how ‘Code Red’ activation correlates with mortality. Another limitation is that the comparison of the 2015 and 2018 data, to assess whether the change in BCSH guidelines affected the success of the ‘Code Red’ protocol, was retrospective. A prospective study with defined data collection points, including functional outcome, would provide more data to ascertain if there is a correlation between ‘Code Red’ activation and mortality.

Although none of the patients in the 2018 cohort weighed >85kg, the BCSH 2015 guidelines recommend dosing FFP based on weight (15–20ml/kg), meaning patients administered with a standard Pack A would be under-dosed with FFP. There was no field in which to record patient weight on the previous ‘Code Red’ paper forms and this has now been changed. As such, we have adapted the protocol to issue FFP based on weight.

There is also discussion about predictors for massive blood loss. Thromboelastometry and thromboelastography are two point-of-care tests that monitor coagulation. A meta-analysis found that use of these two measures during blood loss management led to a fall in blood transfusion as well as a lower risk of re-exploration surgery indicated by postoperative haemorrhage (Deppe et al 2016). These results were repeated when such testing was combined with coagulation factor administration (Görlinger 2011) and integrated within a management algorithm (Karkouti et al 2004).

Finally, establishing a universal definition of massive blood loss may improve protocols worldwide by enabling systematic reviews of papers describing potential developments in the management of perioperative blood loss, which for now are limited by the range of definitions and criteria of massive blood loss. It may also increase preparedness through opening the possibility of predictive tool development and clinicians’ confidence to engage protocols.

To conclude, the massive blood loss protocol ‘Code Red’ at the specialist cardiac unit continues to improve towards meeting the standards set in the BCSH 2006 and 2015 guidelines while delivering often-personalised care, and this preparedness may mean that fewer instances of massive blood loss require activation of ‘Code Red’ protocol. We highlight the need for thorough record-keeping and have adapted the ‘Code Red’ paper forms such that FFP be given according to weight, in line with BCSH 2015 guidelines. We encourage the establishment of a universal definition of massive blood loss and investigation into ways that clinicians may predict this.

## Declarations

### Guarantor

MT.
